# Sema3C signaling is an alternative activator of the canonical WNT pathway in glioblastoma

**DOI:** 10.1038/s41467-023-37397-w

**Published:** 2023-04-20

**Authors:** Jing Hao, Xiangzi Han, Haidong Huang, Xingjiang Yu, Jiankang Fang, Jianjun Zhao, Richard A. Prayson, Shideng Bao, Jennifer S. Yu

**Affiliations:** 1 Center for Cancer Stem Cell Biology, Department of Cancer Biology, Lerner Research Institute, Cleveland Clinic, 9500 Euclid Avenue, Cleveland, OH 44195 USA; 2 Department of Anatomic Pathology, Tomsich Pathology and Laboratory Medicine Institute, Cleveland Clinic, 9500 Euclid Avenue, Cleveland, OH 44195 USA; 3 Burkhardt Brain Tumor and Neuro-Oncology Center, Neurological Institute, Cleveland Clinic, 9500 Euclid Avenue, Cleveland, OH 44195 USA; 4 Department of Radiation Oncology, Taussig Cancer Institute, Cleveland Clinic, 9500 Euclid Avenue, Cleveland, OH 44195 USA

**Keywords:** Cancer stem cells, CNS cancer

## Abstract

The Wnt pathway is frequently dysregulated in many cancers, underscoring it as a therapeutic target. Wnt inhibitors have uniformly failed in clinical trials. Here, we report a mechanism of WNT pathway activation through the Semaphorin 3 C neurodevelopmental program in glioma stem-like cells. Sema3C directs β-catenin nuclear accumulation in a Rac1-dependent process, leading to transactivation of Wnt target genes. Sema3C-driven Wnt signaling occurred despite suppression of Wnt ligand secretion, suggesting that Sema3C drives canonical Wnt signaling independent of Wnt ligand binding. In a mouse model of glioblastoma, combined depletion of Sema3C and β-catenin partner TCF1 extended animal survival more than single target inhibition alone. In human glioblastoma, Sema3C expression and Wnt pathway activation were highly concordant. Since Sema3C is frequently overexpressed in glioblastoma, Sema3C signaling may be a significant mechanism of resistance to upstream Wnt pathway inhibitors. Dual targeting of Sema3C and Wnt pathways may achieve clinically significant Wnt pathway inhibition.

## Abstract (web summary)

Wnt signaling is dysregulated in glioblastoma (GBM). Here the authors show that Semaphorin 3C drives Wnt signaling through Rac1-dependent β-catenin nuclear accumulation and that dual blockade of Semaphorin 3C and Wnt pathway reduces the growth of GBM in vivo.

## Introduction

The Wnt pathway is a fundamental developmental program that is deregulated in numerous cancers^1^. In the canonical pathway, Wnt ligands bind to their receptors, Frizzled and LRP, to inhibit the β-catenin destruction complex, ultimately leading to β-catenin translocation to the nucleus. Nuclear β-catenin then binds to the TCF/LEF transcription complex to drive transcription of Wnt target genes that can drive cancer progression.

Glioblastoma (GBM) is a lethal primary brain tumor that is refractory to standard treatment^2^. In GBM, Wnt signaling is frequently dysregulated, particularly in the glioma cancer stem-like cell (GSC) population, where it plays a critical role in stem cell maintenance^3–5^ and therapeutic resistance^6,7^. Wnt signaling can also facilitate interactions between glioma stem or progenitor cells with other cell types within the tumor microenvironment to facilitate invasion and further confer resistance to treatment^8–10^. In preclinical models of GBM, inhibition of the Wnt pathway itself has minimal benefits in terms of tumor control, but rather indirectly sensitizes tumors to chemotherapy^10,11^.

While mutations in the Wnt pathway have been identified in multiple cancers including the primary brain cancer medulloblastoma^12^, no mutations in *APC* (*Adenomatous Polyposis Coli*) or *CTNNB1* (the gene encoding β-catenin) have been identified in GBM^13^. Rather, overexpression of Wnt ligands, or mutational or epigenetic dysregulation of key pathway regulators are more frequently found^9,14–18^.

Numerous clinical trials have investigated Wnt inhibitors in a variety of cancers^19^. Despite these intense efforts, however, Wnt inhibitors have failed in the clinic^19^. Considering that most Wnt inhibitors target either the ligand or receptor^19,20^, we hypothesized that GSCs have evolved mechanisms to bypass signaling initiated by Wnt ligand-receptor interactions. Defining these resistance pathways may lead to improved tumor control.

The Semaphorin pathway is a neurodevelopmental program that directs the patterning of the nervous and cardiovascular systems^21–24^ and is frequently dysregulated in GBM and other cancers^23,25–30^. We previously reported that the secreted protein Semaphorin 3C (Sema3C) and its receptors are overexpressed in ~85% of GBM and are preferentially utilized by GSCs but not their normal counterparts, neural progenitor cells^25^. Sema3C binding to its Neuropilin-Plexin receptor complex promotes GSC survival and invasion^25,31^. As a secreted protein, Sema3C facilitates communication between neighboring GSCs to promote tumor progression, thereby expanding their population and reinforcing their malignant phenotype collectively.

Sema3C-mediated glioma progression is dependent on activation of the small GTPase Rac1^25^. In embryonic development, Rac1 has been implicated in the regulation of β-catenin translocation from the cytoplasm to the nucleus^32^. We therefore hypothesized that Sema3C-Rac1 signaling may similarly promote β-catenin nuclear localization, effectively bypassing Wnt ligand-receptor interaction and driving downstream canonical Wnt signaling in GSCs. Here, we report that the Sema3C pathway acts as an alternate canonical Wnt pathway activator by driving β-catenin nuclear translocation and conferring resistance to upstream Wnt pathway inhibition. Our data support a therapeutic strategy of targeting of both pathways to improve GBM control.

## Results

### Combined inhibition of Wnt and Sema3C pathways improves survival

Previous studies in preclinical mouse models of GBM suggest minimal if any survival benefit with Wnt inhibition alone despite overwhelming data of improved tumor control in vitro^8,13^. The Porcupine inhibitor LGK974 blocks Wnt secretion and is under clinical investigation^33–35^. Since GSCs are more resistant to therapeutic agents^36,37^, we first tested the sensitivity of GSCs to LGK974. We found that the half maximal effective dose (EC50) of LGK974 is about 6.5 µM in GSCs (Supplementary Fig. 1a). Treatment of GSCs with LGK974 reduced GSC self-renewal and viability in a dose-dependent manner, demonstrating the sensitivity of GSCs to this specific Wnt pathway inhibitor (Supplementary Fig. 1b).

**Fig. 1:**
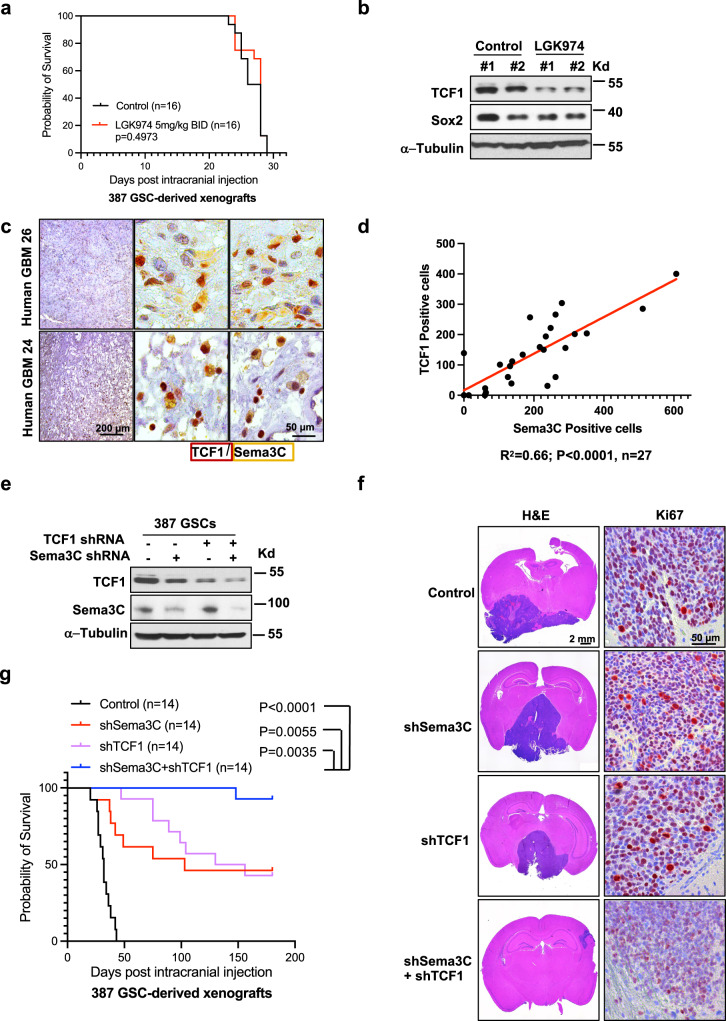
Combined inhibition of Wnt and Sema3C pathways improves survival in a mouse model of GBM. **a** Kaplan–Meier curve of GBM model. 14 days after NSG mice were intracranially injected with 387 GSCs, they were treated with either vehicle (*n* = 16, median survival 27 days) or LGK974 (*n* = 16, median survival 28 days) 5 mg/kg twice a day for 14 days by oral gavage (Log-rank test *p* = 0.4973). **b** Immunoblot of TCF1 expression in GBM tissue of representative control or LGK974 treated animals in (**a**) (representative 2 samples from each group were shown out of 9 samples analyzed from each group). Sox2 and α-Tubulin were used as loading controls. **c** Multiple antigen immunohistochemistry staining of TCF1 (red, nucleus) and Sema3C (yellow, cytosol) in human GBM samples (representative pictures from 27 samples were shown). Left: 10x objective view with scale bar 200 μM. Right: 63x oil objective view with scale bar 50 μM. **d** Scatterplot and linear regression analysis of TCF1 and Sema3C positive stained cells in human GBM (*n* = 27 GBM samples, simple linear regression test, *R*^2^ = 0.66, *p* < 0.0001, Slope = 0.6034, 95% confidence interval 0.43 to 0.78). **e** Western blots of TCF1 and Sema3C after knockdown of TCF1, Sema3C, or both in 387 GSCs used in orthotopic xenograft model in figure panels (**f**) and (**g**). **f** Left panels: H&E staining of mouse brain panorama image. Scale bar 2 mm. Right panels: Ki67 immunohistochemistry staining in shNT, shSema3C, shTCF1, or shSema3C plus shTCF1 knockdown tumors. Scale bar 50 μM. Representative images of 387 GSC-derived xenograft tumor samples of euthanized animals (as in C) are shown (one sample from each group out of 14 was shown). **g** Kaplan–Meier curve of 387 GSC-derived orthotopic xenografts expressing shNT (median survival, 32 days), shSema3C (median survival, 103 days), shTCF1 (median survival, 143 days) or shSema3C + shTCF1 double knockdown (median survival not reached) (*n* = 14 for each group). Log-Rank test, each group compared with control *p* < 0.0001; shSema3c vs. shTCF1, *P* = 0.7461; shSema3C vs. shSema3C + shTCF1, *p* = 0.0055; shTCF1 vs. shSema3C + shTCF1, *p* = 0.0035. Source data are provided as a Source data file.

Considering that many drugs are unable to pass through the blood-brain-barrier and blood-tumor-barrier, we first asked whether LGK974 can engage its target in a GSC-derived orthotopic mouse model of GBM. After confirming tumor growth by bioluminescence imaging, mice were randomized to receive LGK974 or placebo control by oral gavage at two different dose levels (Supplementary Fig. 1c). The higher dose of 10 mg/kg twice a day induced diarrhea in all of the animals after 10 days of treatment, consistent with known intestinal toxicity of Wnt inhibitors^38^, and led to premature death. Mice tolerated the lower dose (5 mg/kg twice a day) without clinically significant toxicity. We expanded our studies to larger numbers of mice (Fig. 1a). Analysis of the tumors from treated animals showed a significant reduction in the expression of the Wnt target gene TCF1^39^ (Fig. 1b). These data suggest that at a well-tolerated dose, LGK974 can indeed pass the blood-brain-barrier and blood-tumor-barrier to engage its target. However, we did not see any survival benefit (Fig. 1a, *p* = 0.4973) even though TCF1 expression was reduced (Fig. 1b) in tumors of treated animals. Our data suggest that blocking Wnt secretion alone is insufficient to reduce tumor growth in vivo and that other pathways contribute to resistance.

Cancer stem cells have evolved mechanisms to reactivate developmental pathways to ensure their survival and maintenance^40^. We next assessed whether other signaling pathways that are often deregulated in GBM, in particular in GSCs, can act as a driver of canonical WNT pathway. Since Sema3C is overexpressed in the overwhelming majority (>85%) of GBM and Sema3C signaling is frequently activated in GSCs^25^, we assessed the possibility that Sema3C could regulate canonical Wnt signaling.

To this end, we first investigated the expression pattern of TCF1, the Wnt target gene and β-catenin binding partner, and Sema3C in human GBM samples (Fig. 1c and Supplementary Fig. 1d, e). By dual immunohistochemical staining, we found that the expression of both proteins is highly correlated (Fig. 1c, d; Pearson correlation coefficient, *r* = 0.82, *R*^2^ = 0.66, *p* < 0.0001), suggesting that they may function in the same signaling axis.

To determine potential interactions between Sema3C and Wnt signaling, we used a genetic approach. We established tumors in which Sema3C, TCF1, or both Sema3C and TCF1 were knocked down. In two different models, knockdown of either Sema3C or TCF1 alone reduced tumor growth and extended animal survival compared to control animals (Fig. 1e–g, Supplementary Fig. 1f, g). Silencing both Sema3C and TCF1 resulted in the greatest tumor control and longest animal survival. Ki67 staining was markedly reduced in tumors in which Sema3C and TCF1 were silenced, supporting a reduction in tumor cell proliferation (Fig. 1f). Together, these data support that inhibition of both Sema3C and Wnt pathways synergize to improve survival more than single pathway inhibition alone.

### Sema3C is indispensable for the self-renewal of GSCs

**Fig. 2:**
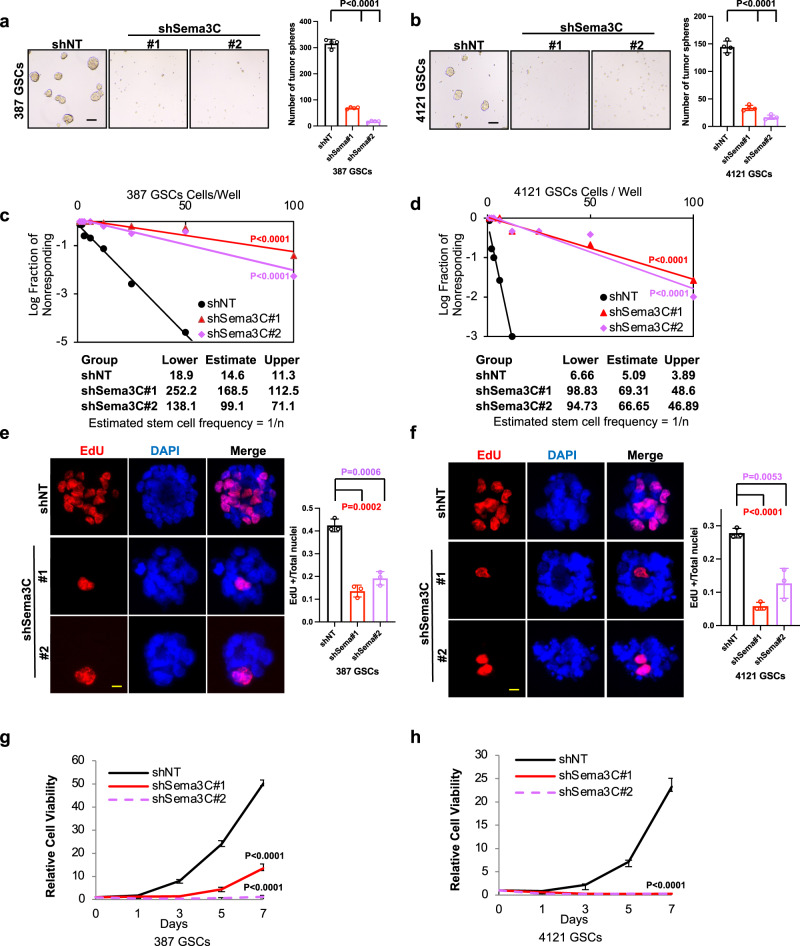
Sema3C promotes GSC self-renewal. **a**, **b** Left panels: representative tumorsphere images of 387 (**a**) and 4121 (**b**) GSCs after control shNT, shSema3C#1, and shSema3C#2 knockdown. Scale bar 100 μM. Right panels: quantification of tumorspheres after Sema3C knockdown (*n* = 4 biological replicates in each group, compared with control unadjusted *p* < 0.0001, error bars: S.D. Scale bar 100 μM). **c**, **d** In vitro extreme limiting dilution assay in Sema3C knockdown 387 GSCs (**c**) and 4121 GSCs (**d**). Tables show estimated stem cell frequencies in control shNT, shSema3C#1, and shSema3C#2 knockdown GSCs with 95% confidence intervals (*n* = 24 replicates in each dose, compared with control unadjusted *p* < 0.0001, error bars: S.D.). **e**, **f** Left panels: representative immunofluorescence images of EdU incorporation assay in 387 (**e**) and 4121 (**f**) GSCs after control shNT, shSema3C#1, and shSema3C#2 knockdown. Right panels: quantification of EdU^+^ cells over total cells (EdU in red, DAPI in blue, *n* = 3 independent experiments, 387 GSCs control vs. shSema3C#1 unadjusted *p* = 0.0002; control vs. shSema3C#2 unadjusted *p* = 0.0006. 4121 GSCs control vs. shSema3C#1 unadjusted *p* < 0.0001; control vs. shSema3C#2 unadjusted *p* = 0.0053, error bars: S.D. Scale bar 50 μM). **g**, **h** Cell viability of 387 and 4121 GSCs after Sema3C knockdown (*n* = 6 biological replicates for each group, compared with control unadjusted *p* < 0.0001, error bars: S.D.). Source data are provided as a Source data file.

Since the Wnt pathway facilitates stem cell self-renewal, and our study suggests a link between Sema3C and Wnt pathways, we investigated the functional role of Sema3C in GSC self-renewal. We assessed tumorsphere formation and performed in vitro extreme limiting dilution assays using GSC lines in which Sema3C was silenced. Sema3C knockdown (shSema3C) reduced tumorsphere formation (Fig. 2a, b) and reduced self-renewal capacity compared to control non-targeting knockdown (shNT) (Fig. 2c, d). The frequency of GSCs was reduced 6- to 13-fold after silencing Sema3C, depending on the cell line (Fig. 2c, d). In addition, Sema3C knockdown significantly reduced proliferation, as evidenced by reduced incorporation of the thymidine analog ethynyldeoxyuridine (EdU) into DNA (Fig. 2e, f). This reduction in proliferation contributed to a decline in cell number after Sema3C knockdown (Fig. 2g, h). These data suggest that Sema3C plays an essential role in GSC self-renewal.

### Sema3C signaling regulates the Wnt pathway

**Fig. 3:**
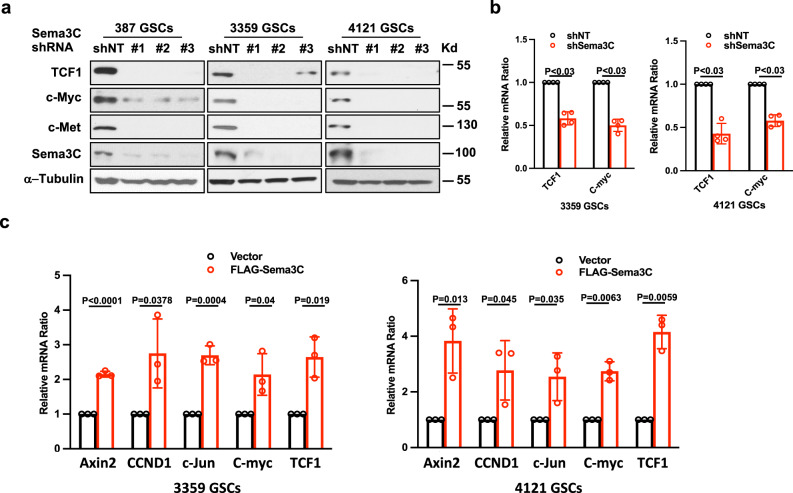
Sema3C regulates Wnt pathway. **a** Western blots of TCF1, c-Myc, and c-Met proteins after Sema3C knockdown. Western blots were repeated at least twice. **b** qRT-PCR of TCF1 and C-myc in 3359 and 4121 GSCs after Sema3C knockdown (*n* = 4 independent experiments, Mann Whitney U-test *p* < 0.03, error bars: S.D.). **c** qRT-PCR of Axin2 (3359 GSCs *p* < 0.0001; 4121 GSCs *p* = 0.013), CCND1 (3359 GSCs *p* = 0.0378; 4121 GSCs *p* = 0.045), c-Jun (3359 GSCs *p* = 0.0004; 4121 GSCs *p* = 0.035), C-myc (3359 GSCs *p* = 0.04; 4121 GSCs *p* = 0.0063) and TCF1(3359 GSCs *p* = 0.019; 4121 GSCs *p* = 0.0059) in GSCs expressing FLAG vector or FLAG-Sema3C (*n* = 3 independent experiments, Two-tailed T-test, error bars: S.D.). Source data are provided as a Source data file.

We next tested the effects of Sema3C on canonical Wnt signaling. Expression of key Wnt target genes including TCF1 and c-Myc were down-regulated at the mRNA and protein levels when Sema3C was silenced (Fig. 3a, b, Supplementary Fig. 2a). We then overexpressed FLAG-tagged full-length Sema3C in GSCs. Overexpression of Sema3C led to increased expression of multiple Wnt target genes including Axin2, CCND1, c-Jun, C-myc, and TCF1 at the mRNA level (Fig. 3c). Changes in expression of a subset of these targets were confirmed at the protein level (Supplementary Fig. 2b). Together, these data suggest that Sema3C can regulate the canonical Wnt pathway.

### Sema3C regulates β-catenin nuclear translocation

**Fig. 4:**
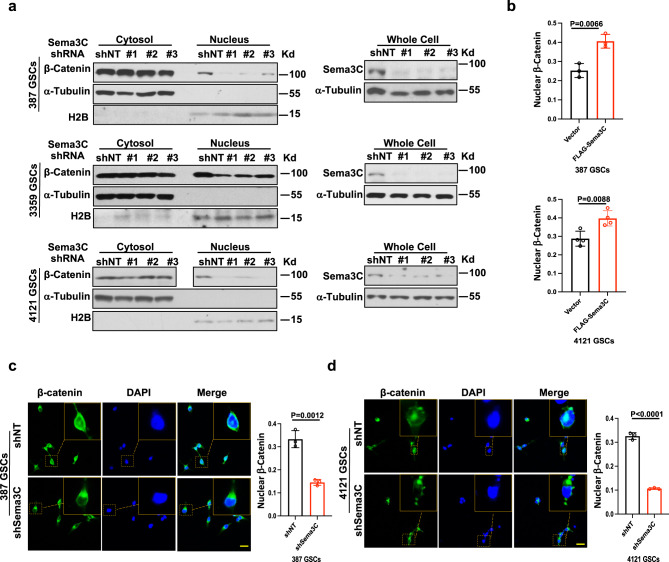
Sema3C promotes β-catenin nuclear localization. **a** Western blots of β-catenin in cytosolic and nuclear fractions (left) of GSCs after Sema3C knockdown (right). Western blots were repeated at least twice. **b** Quantification of nuclear β-catenin positive cells of GSCs expressing FLAG vector or FLAG-Sema3C (*n* = 3 independent experiments in 387 GSCs Two-tailed T-test *p* = 0.0066, error bars: S.D.; *n* = 4 independent experiments in 4121 GSCs, Two-tailed T-test *p* = 0.0088, error bars: S.D.). **c**, **d** Left panels: representative immunofluorescence images of β-catenin (green) in 387 (**c**) and 4121 (**d**) GSCs after control shNT and shSema3C knockdown. Nuclei were counterstained with DAPI (blue). Right panels: quantification of nuclear β-catenin positive cell fraction (*n* = 3 independent experiments, Two-tailed T-test, *p* = 0.0012 (387 GSCs); *p* < 0.0001 (4121 GSCs) error bars: S.D. Scale bar 50 μM). Source data are provided as a Source data file.

In canonical Wnt signaling, translocation of β-catenin into the nucleus is a critical event that leads to transactivation of Wnt target genes^1^. We therefore investigated the role of Sema3C in regulating β-catenin nuclear localization. We performed cellular fractionation assays to separate cytosolic and nuclear fractions of GSCs, followed by immunoblotting of β-catenin. The nuclear fraction of β-catenin was significantly reduced when Sema3C was silenced in four different GSC lines (Fig. 4a, Supplementary Fig. 3). We next complemented these fractionation studies with immunofluorescence imaging of β-catenin. Knockdown of Sema3C in GSCs reduced the fraction of cells exhibiting nuclear β-catenin by at least 50% (Fig. 4c, d). Conversely, overexpression of Sema3C led to an increase in cells with β-catenin nuclear localization (Fig. 4b). These data strongly suggest that Sema3C regulates the subcellular localization of β-catenin.

### Activation of Rac1 by Sema3C facilitates β-Catenin nuclear translocation

**Fig. 5:**
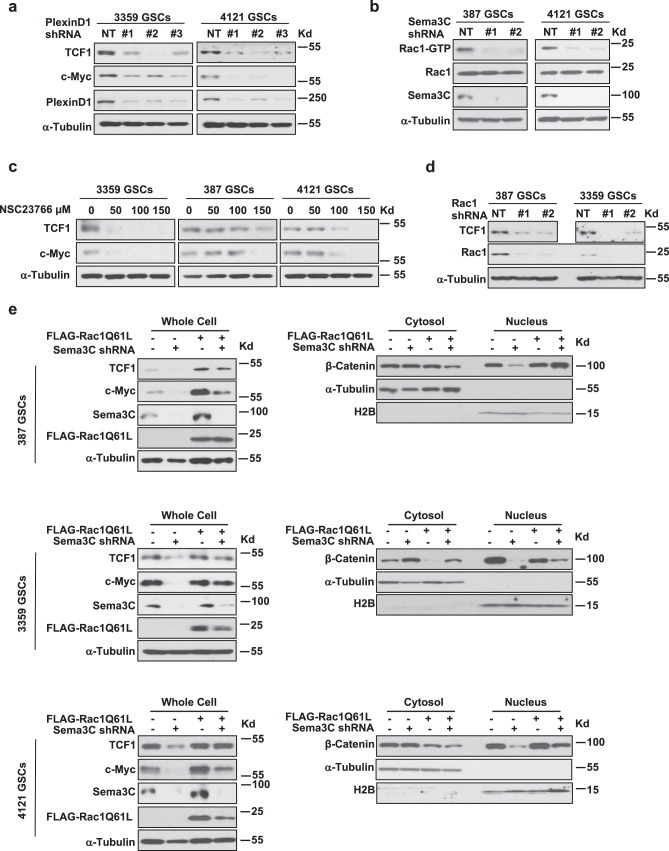
Sema3C induces nuclear β-catenin shuttling via Rac1 activation. **a** Western blots of TCF1 and c-Myc proteins after PlexinD1 knockdown in GSCs. **b** Western blots of Rac1-GTP and Rac1 after Sema3C knockdown in GSCs. **c** Western blots of TCF1 and c-Myc in NSC23766-treated GSCs. **d** Western blots of TCF1 after Rac1 knockdown in GSCs. **e** Western blots of TCF1 and c-Myc in GSCs expressing FLAG-Rac1-Q61L in the setting of Sema3C knockdown. Immunoblots using whole cell lysates (left) and cytosol and nuclear fractions (right) are shown. Source data are provided as a Source data file. Western blots were repeated at least twice.

Sema3C binds to the NRP1/PlexinD1 receptor complex in GSCs^25^. The PlexinD1 co-receptor functions in signal transduction and is essential in GSC maintenance. We reasoned that silencing PlexinD1 would phenocopy the effects of Sema3C knockdown on Wnt signaling. Indeed, we observed a reduction in TCF1 and c-Myc expression in three independent GSCs in which PlexinD1 was silenced (Fig. 5a).

We previously demonstrated that Rac1 is activated upon Sema3C binding to its receptor complex, and Rac1 is an essential mediator of Sema3C-dependent glioblastoma progression^25^. The small GTPase Rac1 can regulate the Wnt pathway by facilitating β-catenin nuclear translocation^32^. Considering the effects of Sema3C on GSC self-renewal, we next assessed the potential convergence of Sema3C and canonical Wnt signaling in GSCs as mediated through Rac1. We first silenced Sema3C and assessed active Rac1 by Rac1-GTP pull-down assays. Rac1-GTP was significantly reduced in GSCs in which Sema3C was silenced (Fig. 5b), consistent with our previous findings^25^.

We next tested the ability of Rac1 to regulate Wnt signaling. GSCs were treated with increasing concentrations of the Rac1 inhibitor NSC23766^41^. After inhibition of Rac1, TCF1 and c-Myc expression were reduced in a dose-dependent manner, phenocopying Sema3C knockdown (Fig. 5c). We observed a similar reduction of TCF1 protein expression when we silenced Rac1 in GSCs (Fig. 5d). These studies suggest that Rac1 mediates Sema3C-induced Wnt signaling.

We next tested the ability of constitutively active Rac1 (Rac1-Q61L)^42^ to restore the expression of TCF1 and c-Myc, and nuclear localization of β-catenin in the setting of Sema3C knockdown. As expected, active Rac1 rescued TCF1 and c-Myc expression (Fig. 5e, left) and increased nuclear β-catenin in all three GSC lines tested (Fig. 5e, right). These data support that Sema3C can regulate the canonical Wnt pathway through activation of Rac1.

### High Sema3C expression drives canonical Wnt signaling despite inhibition of Wnt ligand secretion

**Fig. 6:**
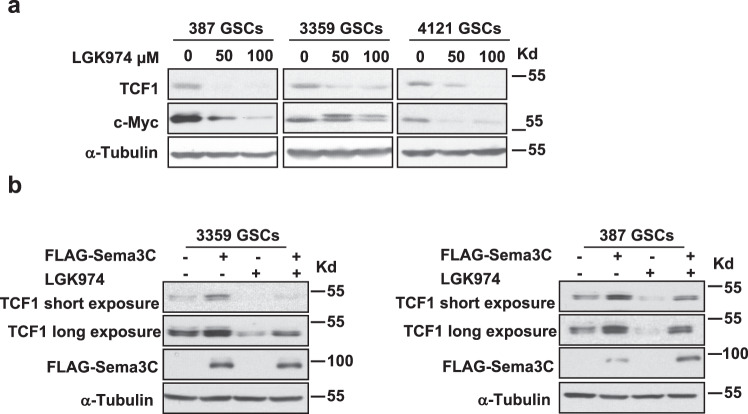
Sema3C confers resistance to Wnt inhibition. **a** Western blots of TCF1 and c-Myc in GSCs treated with Porcupine inhibitor LGK974. Cells were treated with LGK974 or vehicle control for 24 h. **b** Western blots of TCF1 in LGK974 treated GSCs expressing FLAG vector or FLAG-Sema3C. Cells were treated with LGK974 100 μM or vehicle control for 24 h. Source data are provided as a Source data file. Western blots were repeated at least twice.

Our data suggest that Sema3C can drive canonical Wnt signaling by facilitating ß-catenin nuclear translocation irrespective of Wnt ligand-receptor interaction. To test this possibility, we performed Sema3C overexpression studies in the presence of a Wnt pathway inhibitor. Treatment of GSCs with the Porcupine inhibitor, LGK974, reduced expression of TCF1 and c-Myc expression in a dose-dependent manner (Fig. 6a). We next overexpressed FLAG-tagged Sema3C in GSCs followed by treatment with LGK974. Sema3C overexpression rescued LGK974-mediated reduction in TCF1 expression (Fig. 6b). Together, these data support that high levels of Sema3C signaling can drive canonical Wnt signaling to bypass Wnt ligand inhibition.

### Combined Sema3C and Wnt pathway inhibition improve GBM control

**Fig. 7:**
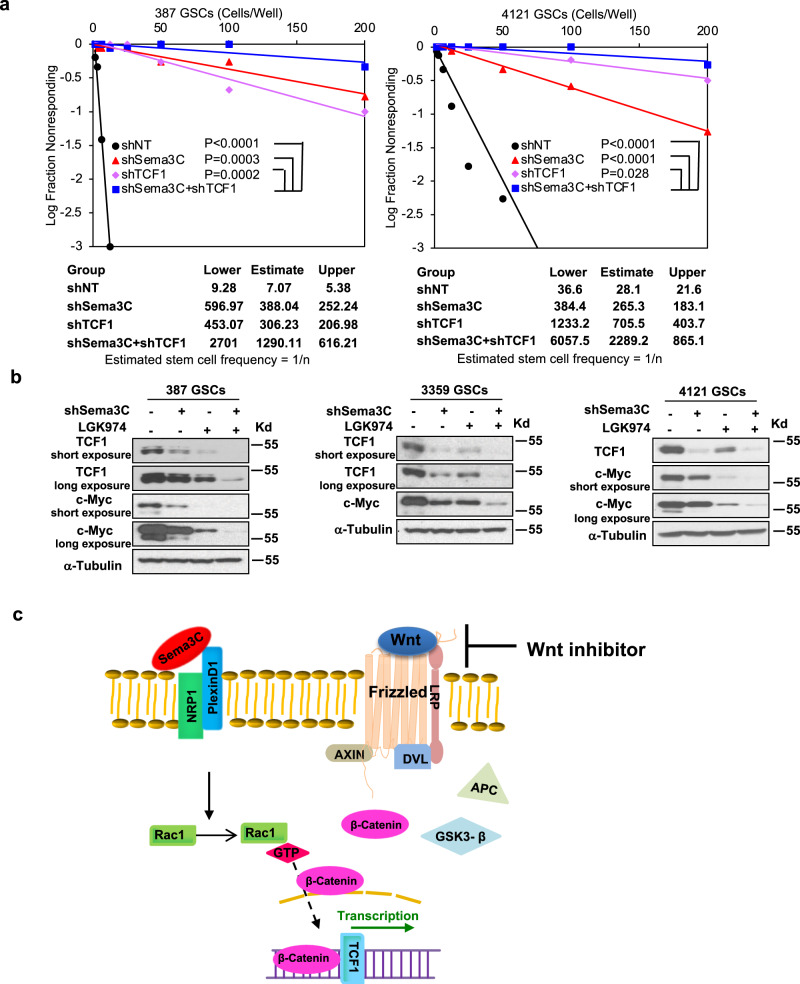
Improved GSC and tumor control with combined Sema3C and Wnt pathway inhibition. **a** In vitro extreme limiting dilution assay in shSema3C, shTCF1 or both knockdowns. Tables below show estimated stem cell frequencies in control shNT, shSema3C, shTCF1, and shSema3C + shTCF1 knockdown GSCs (*n* = 8 technical replicates in each dose, at least three biological replicates, ELDA test, comparing to shSema3C + shTCF1 knockdown GSCs, 387 GSCs: shNT *p* < 0.0001; shSema3C *P* = 0.0003; shTCF1 *p* = 0.0002; 4121 GSCs: shNT *p* < 0.0001; shSema3C *p* < 0.0001; shTCF1 *p* = 0.028). **b** Western blots of TCF1 and c-Myc in shSema3C knockdown GSCs treated with LGK974 100 μM or vehicle control for 24 h. Western blots were repeated at least twice. **c** Model of Sema3C regulation of the Wnt pathway in GSCs. Despite upstream Wnt pathway inhibition, Sema3C binds to the Neuropilin 1 (NRP1)—PlexinD1 receptor complex pathway to activate Rac1. Active Rac1 (Rac1-GTP) facilitates β-catenin nuclear translocation to drive Wnt target gene transcription. Source data are provided as a Source data file.

GSCs are dependent on the Sema3C pathway and Wnt signaling for their maintenance. We reasoned that inhibition of both Sema3C and Wnt pathways would achieve better GSC control than inhibition of either pathway alone. To this end, we silenced Sema3C and TCF individually or together. Silencing both Sema3C and TCF1 led to the greatest reduction in GSC self-renewal capacity compared to single knockdown in limiting dilution assays, suggesting synergy with dual inhibition (Fig. 7a). Similarly, combined Sema3C knockdown with the Wnt pathway inhibitor LGK974 disrupted Wnt signaling more than single pathway treatment alone (Fig. 7b). These in vitro data support our animal studies that dual inhibition of Sema3C and Wnt pathways reduces GSC self-renewal and GSC-mediated tumorigenesis (Fig. 1g, Supplementary Fig. 1f).

Based on our findings, we propose a model by which Sema3C can drive Wnt signaling irrespective of Wnt ligand binding to its receptor (Fig. 7c). Sema3C signaling activates Rac1 to facilitate β-catenin translocation into the nucleus to initiate downstream Wnt target gene expression. Therefore, GSCs can bypass Wnt ligand-receptor interaction and instead directly engage downstream Wnt pathway effectors to promote their self-renewal and facilitate tumor growth.

## Discussion

Inhibition of the Wnt signaling pathway is an attractive therapeutic approach given its frequent deregulation in a wide range of tumor types. However, resistance mechanisms have held Wnt inhibitors at bay. The precise mechanisms driving this resistance are unclear. We have identified a potentially prevalent mechanism by which GSCs are refractory to upstream Wnt pathway inhibition.

Our studies support that Sema3C helps to maintain GSC self-renewal and drive tumor progression by promoting Wnt signaling. Since Sema3C is overexpressed in 85% of GBM^25^, our data suggest that Sema3C signaling may contribute to resistance to Wnt inhibitors in many patients. In human GBM, Sema3C and TCF1 expression are strongly concordant. In GSCs, Sema3C signaling through Rac1 activation leads to nuclear accumulation of β-catenin and subsequent transactivation of Wnt target genes. Importantly, we found that Sema3C-dependent Wnt signaling can occur despite suppression of Wnt ligand secretion, suggesting that Sema3C drives Wnt signaling independent of Wnt ligand binding.

These findings have significant clinical implications. Efforts to develop Wnt pathway or Rac1 inhibitors have been unfruitful in GBM^43,44^. This may in part be due to activation of multiple upstream pathways that regulate key effectors. Accumulating evidence suggests that translocation of β-catenin is deregulated in GBM. The transcription factor FoxM1 binds to and facilitates β-catenin nuclear translocation^45,46^. Inactivating mutations of FAT1, a cadherin-like protein that binds β-catenin and sequesters it in the cytoplasm, is found in ~20% of GBM^17^. Our data now support that the Sema3C-Rac1 axis may serve as an alternate and frequently used mechanism to drive β-catenin translocation into the nucleus, ultimately to facilitate canonical Wnt signaling.

The toxicities of current Wnt inhibitors preclude further dose escalation. The Sema3C/PlexinD1/Rac1/β-catenin signaling axis may represent a therapeutic vulnerability to inhibit the Wnt pathway in GSCs. Our data support that Sema3C signaling is indispensable for GSC maintenance but not that of neural progenitor cells^25^. Furthermore, Sema3C knockout mice do not exhibit overt gastrointestinal signs^47^, a dose-limiting toxicity of many Wnt inhibitors^19^, suggesting that Sema3C is unlikely to regulate Wnt signaling in the gastrointestinal tract. As proof-of-principle, we showed that depletion of both Sema3C and TCF1 prolonged animal survival better than either target alone.

Inhibitors have been developed to target different levels of the Wnt pathway^44^. Most of them are designed to inhibit Wnt ligands or their receptors. We demonstrated that upstream Wnt pathway inhibition alone is insufficient to impact animal survival. Downstream inhibition of TCF1 alone was better, but most animals still succumbed to disease progression. Targeting both Sema3C and TCF1 resulted in sustained tumor control with almost all animals surviving. Our data highlight that dual targeting approaches are needed to inhibit both the canonical Wnt pathway and alternate regulatory pathways, including Sema3C signaling. Overexpression of Sema3C may confer resistance to Wnt inhibitors, not just in GBM, but also in other cancers such as breast and castration-resistant prostate cancers that also utilize both pathways^48,49^. Our study provides a therapeutic approach of targeting the Sema3C pathway to increase the efficacy of Wnt pathway inhibitors.

## Methods

### Cells and cell culture

GSCs were isolated from GBM surgical specimens or mouse xenografts as previously described^25^. Briefly, tumor cells were isolated from fresh surgical samples using the Papain Dissociation System (Worthington Biochemical). Dissociated cells were plated in a Matrigel (Corning) covered culture plate and recovered in stem cell medium (Neurobasal-A medium with B27 supplement, 10 ng/ml EGF and 10 ng/ml FGF) until tumorspheres were observed. Sphere forming cells were sorted by magnetic cell sorting using the surface marker CD133 (Miltenyi Biotec.). CD133 + sphere forming cells were cultured in stem cell medium as described above. These cells were tested by subcutaneous and intracranial transplantation in mice to confirm their tumor forming ability. GBM surgical specimens were collected in compliance with Cleveland Clinic Institutional Review Board-approved protocol. LGK974 and NSC23766 were purchased from Selleckchem (Cat# S7143 and S8031). They were diluted in DMSO as stock solution.

### Immunofluorescence

Immunofluorescent staining of cells was performed as previously described^25^. Briefly, GSCs were plated on 1% Matrigel covered coverslips overnight before fixed with 4% paraformaldehyde (PFA, Sigma-Aldrich) for 10 min. Samples were permeabilized with 0.2% Triton X-100 (Bio-Rad) in PBS for 20 min and blocked with 10% BSA (Sigma-Aldrich) for 60 min at room temperature. Primary antibody incubation was carried out overnight at 4 °C followed by appropriate secondary fluorescently labeled antibodies (Invitrogen) for one hour at room temperature. Nuclei were counterstained with DAPI. Images were acquired using a wide-field fluorescence microscope or SP-5 confocal microscope (Leica).

### Immunohistochemistry

Immunohistochemical staining of tissue sections were performed with an ABC kit using DAB (3,30-Diaminobenzine) substrate (Vector Laboratories SK-4105) or AEC substrate (Millipore 152224, 152226). Two-colored immunohistochemistry was performed following the multiple antigen IHC protocol by Vector Laboratories. Briefly, after deparaffinization, rehydration and antigen retrieval, endogenous enzymes were quenched by BLOXALL reagent (Vector Laboratories). Slides were blocked by Avidin/Biotin blocking reagent (Vector Laboratories, SP-2001) and protein blocking solution (0.5% Casein, 0.5% BSA, and 0.1% Sodium azide in PBS) sequentially. The primary antibody TCF1 (Rabbit, 1:100 diluted in 10% BSA in PBS) was applied to the slides and incubated at 4 °C overnight. HRP conjugated anti-Rabbit secondary antibody (1:200, 1 h at room temperature) incubation was followed by ABC-HRP reagent (Vector laboratories) labeling (1 h at room temperature). AEC reagent was used for TCF1 staining. Second primary antibody Sema3C (Rat, 1:400 diluted in 10% BSA in PBS) was applied after washing off excessive AEC reagent. Repeat secondary (HRP conjugated anti-Rat antibody) antibody and ABC-HRP incubation as in the first primary antibody staining. DAB reagent was used for Sema3C staining. Quantification of Sema3C and TCF1 positive cells were conducted under 63x oil immersed objective fields. Five visual fields were analyzed for each sample. Sema3C and TCF1 positive cells were counted from the same visual fields. The total positive cells from each sample were used in data analysis and plotting and reported as counted cell numbers in all visual fields.

### Cell fractionation and immunoblot

GSC spheres were collected and dissociated with trypsin. After washing with PBS, cell pellets were resuspended in buffer A (10 mM HEPES pH 7.9, 10 mM KCl, 1.5 mM MgCl_2_, 0.34 M sucrose, 10% glycerol, 0.1% Triton X-100, proteinase and phosphatase inhibitors). Cells were lysed on ice for 10 min and centrifuged at 1300 × *g* for 5 min at 4 °C. The supernatants (cytosol fraction) were collected and further centrifuged at 20,000 × *g* for 10 min to clear the debris. The remaining pellets were washed by buffer A for 3 times (1300 g for 5 min at 4 °C) and lysed in RIPA buffer (150 mM NaCl, 1.0% NP 40, 0.5% sodium deoxycholate, 0.1% SDS, 50 mM Tris, pH 8.0, proteinase and phosphatase inhibitors) as nuclear fraction. Whole cell lysates were obtained by dissolving cell pellets in RIPA buffer and the lysate were sonicated and cleared by high-speed centrifuge (20,000 × *g* for 10 min). Protein samples were measured and resolved by SDS-PAGE gel electrophoresis and transferred onto PVDF membranes. Blots were incubated with primary antibodies overnight at 4 °C followed by HRP-conjugated species-specific antibodies (Cell Signaling). Antibodies used are listed below: Sema3C (IB) (Thermo Fisher Scientific, Cat# PA5-24997); Sema3C (IHC) (R&D Systems, Cat#MAB1728); TCF1 (Cell Signaling Technology, Cat#2203); c-Myc (Cell Signaling Technology, Cat#5605); c-Met (Cell Signaling Technology, Cat#8198); β-catenin (BD Transduction Laboratories, Cat#610153); α-Tubulin (Sigma-Aldrich, Cat#T6199-200UL); FLAG (Sigma-Aldrich, Cat#F1804); H2B (Santa Cruz Biotechnology, Cat#sc10808); Rac1 (Cytoskeleton, Cat#ARC03) and Ki-67 (Novocastra, Cat#NCL-Ki67p).

### Cell viability, EC50, and tumor sphere forming assays

For cell viability EC50 and tumor sphere forming assays, 1 × 10^3^ cells were plated into each well of 96-well plates. Cell titers were determined after the indicated number of days using the Cell Titer-Glo Luminescent Cell Viability Assay kit (Promega, G7571). Tumor spheres were counted after 7 days in culture. All data were performed in triplicate and normalized to day 0 and presented as mean ± standard deviation.

### EdU incorporation assay

EdU incorporation assay was performed using Click it EdU cell proliferation kit (Invitrogen, C10339). Briefly, cells were collected after incubation with EdU (10 µM) for 2 h; cells were divided in two parts and click reaction were performed in 1.5 ml centrifuge tubes. One part of the cells was dissociated with trypsin to make single cell suspension for quantification purpose and the other part was left in sphere form for imaging.

### Extreme limiting dilution assay

Cells were plated in 96-well plates at doses ranging from 1 cell per well to 200 cells per well. Each concentration of cells had 8 technical replicates in each dose, at least three biological replicates. After 7 days of culture wells that showed sphere were recorded as positive while wells did not show sphere were recorded as negative. Statistics were performed by extreme limiting dilution assay (ELDA) online tool (http://bioinf.wehi.edu.au/software/elda/).

### DNA constructs and lentiviral transfection

Lentiviral clones expressing NT shRNA, Sema3C, PlexinD1, or TCF1 shRNAs were acquired from Sigma-Aldrich. shRNAs for each gene that displayed high knockdown efficiency (>80% reduction) were used for all related experiments. Construction of constitutively active Rac1-Q61L (Flag-Rac1Q61L) was described before^25^. pCDH-FLAG-Sema3C construct was cloned by inserting Sema3C cDNA into pCDH-puro vector by *BamH1* and *Not1* sites. Viral particles were produced in 293FT cells with the pPACK set of helper plasmids (System Biosciences) in stem cell media. For rescue experiments, GSCs were transduced with Flag-Rac1Q61L lentiviral construct or pCDH-FLAG control vector and allowed to recover for 48 h. Cells were selected by exposure to puromycin for 48 h, and then these cells expressing Flag-Rac1Q61L or FLAG-control were transduced with Sema3C (shSema3C) or control non-targeting (shNT) shRNAs via lentiviral infection. 48 h post infection cells were collected for analysis.

### Orthotopic mouse xenografts

All animal experiments were approved by the Institutional Animal Care and Use Committee at Cleveland Clinic. NSG mice of both sex at 4 to 8 weeks old were used in the experiments. Intracranial transplantation of GSCs to establish GBM xenografts was performed as described^25^. For LGK974 (Selleckchem, Cat#S7143) studies, 2 × 10^4^ GSCs, which stably expressed firefly luciferase, were implanted into the right frontal lobes of NSG mice. Tumor growth was confirmed by bioluminescence imaging on day 11th after tumor transplantation. LGK974 was diluted in DMSO at 50 mg/ml as stock solution. LGK974 was formulated in 0.5% (vol/vol) methyl cellulose. It was administered by oral gavage at 5 mg/kg or 10 mg/kg BID in 200 µl total volume. Control animals were administered with vehicle alone. Animals were treated for 14 days starting 12 days post-intracranial tumor cell transplantation. For the knockdown studies, 48 h after lentiviral infection, 2 × 10^4^ GSCs were transplanted into the right frontal lobes of NSG mice. Animals were maintained until manifestation of neurological signs or for 180 days post-transplantation. Mice were euthanized and necropsied when exhibiting signs of declining neurologic status or performance status. Animals were anesthetized and underwent cardiac perfusion with PBS.

### RNA isolation and real-time PCR

Total RNA was isolated from cultured cells using RNeasy Kit (Qiagen). cDNA was synthesized by reverse transcription and subjected to real-time PCR with human Axin2, CCND1, c-Jun, c-Myc, and TCF1 primers in the presence of Cyber green PCR-Mix (Applied Biosystems). Data were analyzed from three independent experiments and are shown as the mean ± S.D. The following primer pairs were used to detect the mRNA levels of the following genes by RT-qPCR: Human Axin2 (Forward CAACACCAGGCGGAACGAA, Reverse GCCCAATAAGGAGTGTAAGGACT); Human CCND1 (Forward TGGAGCCCGTGAAAAAGAGC, Reverse TCTCCTTCATCTTAGAGGCCAC); Human c-Jun (Forward GAGGGGGTTACAAACTGCAA, Reverse TCTCACAAACCTCCCTCCTG); Human c-Myc (Forward TTTCGGGTAGTGGAAAACCA, Reverse CACCGAGTCGTAGTCGAGGT); Human TCF1 (Forward CTGGCTTCTACTCCCTGACCT, Reverse ACCAGAACCTAGCATCAAGGA); Human β-Actin (Forward AGAAAATCTGGCACCACACC, Reverse AGAGGCGTACAGGGATAGCA).

### Statistics and reproducibility

All grouped data are presented as mean ± S.D. unless otherwise specified. All the western blot data were repeated at least twice. All the other in vitro experiments were repeated at least three times. GraphPad Prism Software (GraphPad Software, Inc.) was used to examine statistical significance with Student’s t test or Mann–Whitney U test as appropriate, log-rank or one-way ANOVA.

### Reporting summary

Further information on research design is available in the Nature Portfolio Reporting Summary linked to this article.

### Supplementary information

Supplementary Information

Reporting Summary

### Source data

Source Data

## Data Availability

All relevant data supporting the key findings of this study are available within the article and its Supplementary Information files. Source data are provided with this paper.
