# Author Correction: Sema3C signaling is an alternative activator of the canonical WNT pathway in glioblastoma

**DOI:** 10.1038/s41467-023-41842-1

**Published:** 2023-09-27

**Authors:** Jing Hao, Xiangzi Han, Haidong Huang, Xingjiang Yu, Jiankang Fang, Jianjun Zhao, Richard A. Prayson, Shideng Bao, Jennifer S. Yu

**Affiliations:** 1 Center for Cancer Stem Cell Biology, Department of Cancer Biology, Lerner Research Institute, Cleveland Clinic, 9500 Euclid Avenue, Cleveland, OH 44195 USA; 2 Department of Anatomic Pathology, Tomsich Pathology and Laboratory Medicine Institute, Cleveland Clinic, 9500 Euclid Avenue, Cleveland, OH 44195 USA; 3 Burkhardt Brain Tumor and Neuro-Oncology Center, Neurological Institute, Cleveland Clinic, 9500 Euclid Avenue, Cleveland, OH 44195 USA; 4 Department of Radiation Oncology, Taussig Cancer Institute, Cleveland Clinic, 9500 Euclid Avenue, Cleveland, OH 44195 USA

**Keywords:** Cancer stem cells, CNS cancer

Correction to: *Nature Communications* 10.1038/s41467-023-37397-w, published online 20 April 2023

In this article the author name Jiankang Fang was incorrectly written as Jiangkang Fang. The original article has been corrected.

