# Supplementary material for: Sema3C signaling is an alternative activator of the canonical WNT pathway in glioblastoma
Source: Nat Commun. 2023 Apr 20;14:2262. doi: 10.1038/s41467-023-37397-w (PMC10119166; doi:10.1038/s41467-023-37397-w)
Supplement: Supplementary file 1 — Supplementary Information [file 41467_2023_37397_MOESM1_ESM.pdf]

Supplementary Fig. 1, related to Fig. 1

a

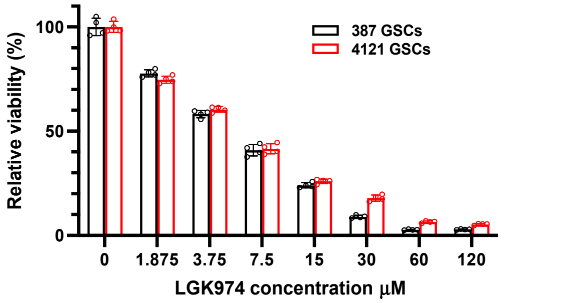

	387 GSCs	4121 GSCs
LGK 974 EC50 (µM)	6.314 ± 0.255	6.581 ± 0.326

b

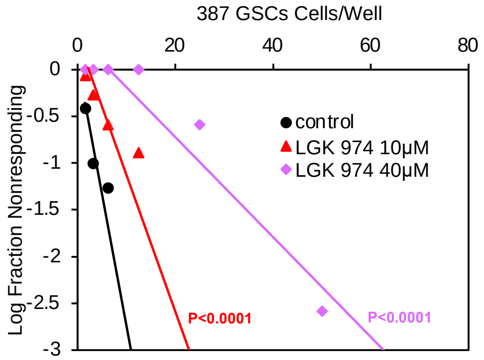

Group	Lower	Estimate	Upper
control	7.63	5.91	4.61
LGK 974 10µM	19.15	14.8	11.46
LGK 974 40µM	60.42	46.59	35.95

c

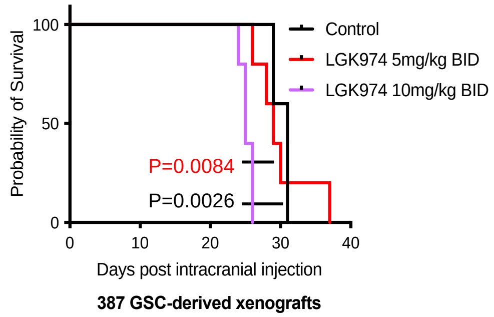

d

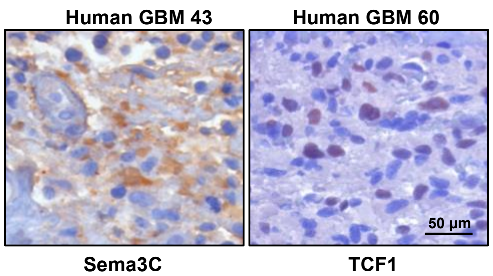

e

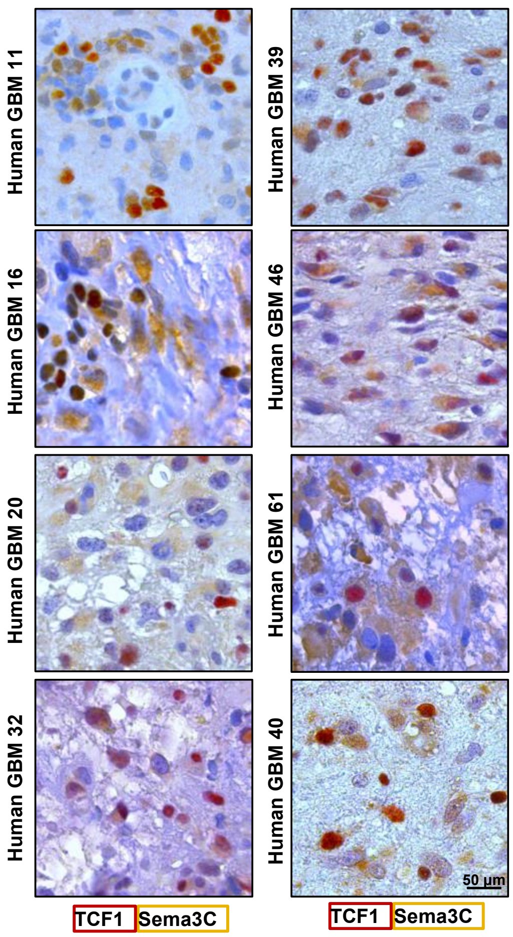

f

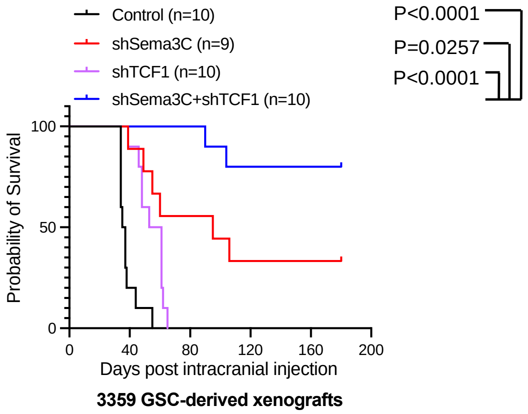

g

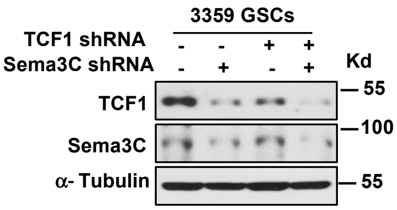

Supplementary Fig.1 related to Fig.1: GSCs are sensitive to LGK974 both in vitro and in vivo.

a, EC50 of LGK974 in GSCs. 387 and 4121 GSCs are treated with escalating doses of LGK974 for 3 days (n=4 replicates in each dose, error bars: S.D.). Cell viability was measured by Cell Titer Glow assays.

b, *in vitro* extreme limiting dilution assay in control, LGK 974 10μM and LGK974 40μM treated 387 GSCs. Table below shows estimated stem cell frequencies in each treatment group with 95% confidence intervals (n=8 technical replicates in each dose, at least three biological replicates, ELDA test, compared with control p <0.0001; LGK974 10μM vs. LGK974 40μM p <0.0001).

c, Kaplan-Meier curve of NSG mice implanted with 387 GSCs treated with vehicle, LGK 974 5mg/kg BID or LGK 974 10mg/kg BID (n=5 for each group). (Log-Rank test, control vs. LGK 974 5mg/kg p=0.5134; control vs. LGK 974 10mg/kg p =0.0026; LGK 974 5mg/kg vs. LGK 974 10mg/kg p=0.0084).

d, Immunohistochemistry of Sema3C (cytoplasm staining) and TCF1 (nuclear staining) in human GBM samples (representative pictures were taken from 5 samples).

e, Multiple antigen immunohistochemistry staining of TCF1 (red, nucleus) and Sema3C (yellow, cytosol) in human GBM samples. All pictures were taken under 63x oil objective view with scale bar 50μM (representative pictures were taken from 27 samples).

f, Kaplan-Meier curve of 3359 GSC-derived orthotopic xenografts expressing shNT (median survival, 36 days; n=10), shSema3C (median survival, 95 days; n=9), shTCF1 (median survival, 57 days; n=10) or shSema3C+shTCF1 double knockdown (median survival not reached; n=10). Log-Rank test, each group compared with control p<0.0001;

shSema3c vs. shTCF1, *p*=0.0353; shSema3C vs. shSema3C+shTCF1, *p*=0.0257; shTCF1 vs. shSema3C+shTCF1, *p*<0.0001.

g, Western blots of TCF1, Sema3C after knockdown of TCF1, Sema3C, or both in 3359 GSCs.

Source data are provided as a Source Data file.

Supplementary Fig. 2, related to Fig. 3

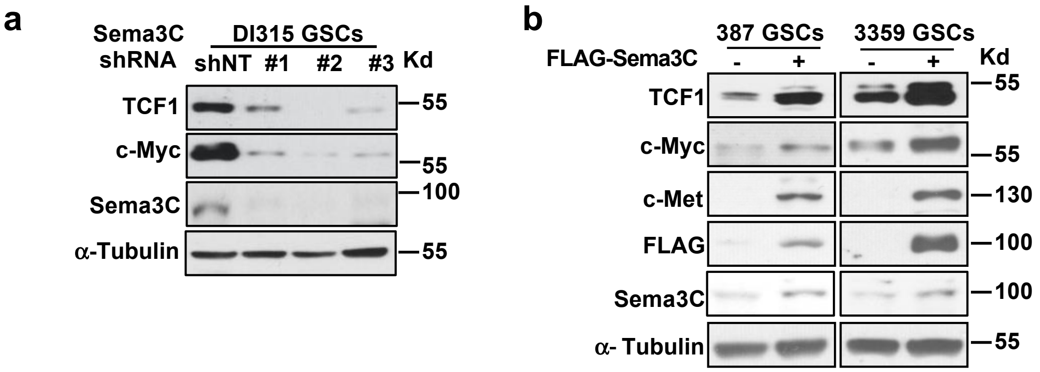

Supplementary Fig.2 related to Fig.3: Sema3C regulates the Wnt pathway.

a, Western blots of TCF1 and c-Myc after Sema3C knockdown in DI315 GSC samples.

b, Western blots of TCF1, c-Myc and c-Met in 387 and 3359 GSCs with FLAG-Sema3C overexpression.

Source data are provided as a Source Data file.

Supplementary Fig. 3, related to Fig. 4

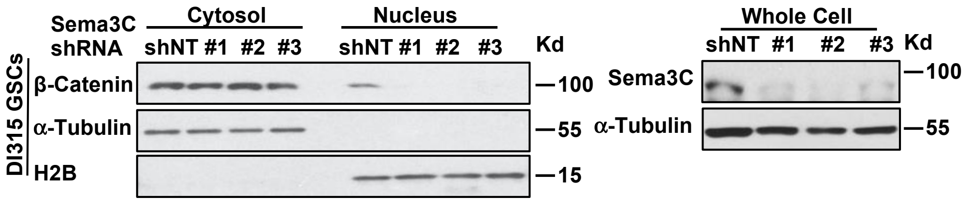

Supplementary Fig.3 related to Fig.4: Sema3C regulates β-catenin nuclear translocation in DI315 GSCs.

Western blots of β-catenin in cytosolic and nuclear fractions (left) in DI315 GSCs after Sema3C knockdown(right).

Source data are provided as a Source Data file.
